# “I feel good when I drink”—detecting childhood-onset alcohol abuse and dependence in a Ugandan community trial cohort

**DOI:** 10.1186/s13034-020-00349-z

**Published:** 2020-10-24

**Authors:** Ingunn Marie Stadskleiv Engebretsen, Joyce S. Nalugya, Vilde Skylstad, Grace Ndeezi, Angela Akol, Juliet N. Babirye, Victoria Nankabirwa, James K. Tumwine

**Affiliations:** 1 Global Mental Health Research Group, Centre for International Health, Department of Global Public Health and Primary Health Care, Faculty of Medicine, University of Bergen, Bergen, Norway; 2 Department of Psychiatry, School of Medicine, College of Health Sciences, Makerere University, Kampala, Uganda; 3 Department of Paediatrics and Child Health, School of Medicine, College of Health Sciences, Makerere University, Kampala, Uganda; 4 School of Public Health, College of Health Sciences, Makerere University, Kampala, Uganda; 5 Centre for Intervention Science in Maternal and Child Health (CISMAC), Centre for International Health, Department of Global Public Health and Primary Health Care, Faculty of Medicine, University of Bergen, Bergen, Norway

**Keywords:** Early onset, Childhood, Alcoholism, Alcohol dependence, Alcohol abuse, Alcohol use disorder, Community cohort, Uganda, MINI-KID, DSM-IV, Child development, Mental health

## Abstract

**Background:**

Alcohol, substance use, and mental health disorders constitute major public health issues worldwide, including in low income and lower middle-income countries, and early initiation of use is an important predictor for developing substance use disorders in later life. This study reports on the existence of childhood alcohol abuse and dependence in a sub-study of a trial cohort in Eastern Uganda.

**Methods:**

The project SeeTheChild—Mental Child Health in Uganda (STC) included a sub-study of the Ugandan site of the study PROMISE SB: Saving Brains in Uganda and Burkina Faso. PROMISE SB was a follow-up study of a trial birth cohort (PROMISE EBF) that estimated the effect that peer counselling for exclusive breast-feeding had on the children’s cognitive functioning and mental health once they reached 5–8 years of age. The STC sub-study (N = 148) used the diagnostic tool MINI-KID to assess mental health conditions in children who scored medium and high (≥ 14) on the Strengths and Difficulties Questionnaire (SDQ) in the PROMISE SB cohort N = (119/148; 80.4%). Another 29/148 (19.6%) were recruited from the PROMISE SB cohort as a comparator with low SDQ scores (< 14). Additionally, the open-ended questions in the diagnostic history were analysed. The MINI-KID comprised diagnostic questions on alcohol abuse and dependence, and descriptive data from the sub-study are presented in this paper.

**Results:**

A total of 11/148 (7.4%) children scored positive for alcohol abuse and dependence in this study, 10 of whom had high SDQ scores (≥ 14). The 10 children with SDQ-scores ≥ 14 had a variety of mental health comorbidities of which suicidality 3/10 (30.0%) and separation anxiety disorder 5/10 (50.0%) were the most common. The one child with an SDQ score below 14 did not have any comorbidities. Access to homemade brew, carer’s knowledge of the drinking, and difficult household circumstances were issues expressed in the children’s diagnostic histories.

**Conclusions:**

The discovery of alcohol abuse and dependence among 5–8 year olds in clinical interviews from a community based trial cohort was unexpected, and we recommend continued research and increased awareness of these conditions in this age group.

*Trial registration* Trial registration for PROMISE SB: Saving Brains in Uganda and Burkina Faso: Clinicaltrials.gov (NCT01882335), 20 June 2013. Regrettably, there was a 1 month delay in the registration compared to the commenced re-inclusion in the follow-up study: https://clinicaltrials.gov/ct2/show/NCT01882335?term=saving+brains&draw=2&rank=1

## Background

The United Nation’s Sustainable Development Goals have made preventing mental ill-health and substance use a public health priority specified in goals 3.4: “By 2030, reduce by one third premature mortality from non-communicable diseases through prevention and treatment and promote mental health and well-being” and 3.5: “Strengthen the prevention and treatment of substance abuse, including narcotic drug abuse and harmful use of alcohol” [1]. Data from the Global Burden of Disease Study 2010 showed that substance use and mental disorders were the leading causes of years lived with disability (YLD) worldwide [2]. Among children and adolescents in low and middle-income countries (LMIC) these conditions accounted for 23.8% of the YLD [3]. It has been estimated that up to 20% of children and adolescents globally suffer from a debilitating mental illness [4, 5] and that 50% of all mental disorders start before age 14 [3, 4]. The World Health Organization (WHO) has estimated that up to 76–85% of all people living with severe mental disorders and 99% of those with past-year substance use disorders in LMIC did not receive treatment [6, 7].

Alcohol consumption in Uganda is among the highest in the world at 26.0 L of alcohol per capita per year in 2016 [8]. At the time of the study the legal regulations from 1960 had not been updated, thus still stating that the legal age for consumption is 18 years, while selling to minors is penalised with a fine not exceeding 500 Ugandan shilling (0.13 USD) and children are allowed to buy on behalf of adults [9]. The Ugandan government has recognised mental illness as a serious public health matter, but service provision remains a major challenge [10]. As a response to the inadequate handling of child and adolescent mental health, the Ministry of Health recently released “Child and Adolescent Mental Health Policy Guidelines” [11] in which substance use in children is recognised, stating that *“alcohol and drug abuse in children and adolescents in Uganda is on the increase although not well researched”* [11].

A family history of substance use is an important predictor for early onset of alcohol use [12–18], and early initiation of alcohol and substance use is an important predictor for later life harmful use [19]. Harmful substance use and mental illness are often co-morbid conditions that share risk factors and outcomes such as school dropout, sexually transmitted diseases, unwanted pregnancies, traffic accidents, violence, and suicide [20–22]. Harmful alcohol use is, according to ICD-10, a diagnosis requiring “actual damage to the mental or physical health of the user” [23]. Further, early exposure to alcohol may affect the developing brain [21]. According to the US National Institute on Alcohol Abuse and Alcoholism, DSM-IV described two distinct disorders, namely alcohol abuse and alcohol dependence, with specific criteria for each. In DSM-5 these were integrated into one—alcohol use disorder—with mild, moderate, and severe sub-classifications [24].

Until now, research on early substance use has focused on adolescents [21, 25, 26] and has not looked at risk factors or the effects of very early use [19]. The WHO has recognised a lack of knowledge about substance use before age 15 [8], making it difficult to address the needs of this group and to plan appropriate interventions. This article reports on the detection of alcohol abuse and dependence in a birth cohort of children aged 5–8 years in Mbale District, Eastern Uganda.

## Methods

### Design, objective, and study site

The current study was part of the SeeTheChild—Mental Child Health in Uganda (STC) project. That project had many components, of which one was an ancillary study to the PROMISE Saving Brains (PROMISE SB) study [27]. The PROMISE SB data collection in 2013–2015 was a follow-up study of a cluster randomised trial cohort promoting exclusive breastfeeding among 2579 mother-infant pairs by peer counsellors in Burkina Faso, Uganda, and South Africa **(**PROMISE-EBF) in the period 2006–2008 and is described elsewhere [28]. The PROMISE SB saw the children in Uganda and Burkina Faso again from 2013 to 2015 when they had reached 5–8 years of age [27].

The Ugandan study site was in Mbale District in Eastern Uganda, 245 km from the capital Kampala. Situated close to the Kenyan border, Mbale Municipality is the main urban trading area in the district, which is predominantly rural and dominated by peasant farming. The Municipality has a regional referral hospital with a psychiatric department.

The STC sub-study included a broader mental health assessment of a selection of children from the PROMISE SB study in Uganda compared to what was assessed in PROMISE SB. This was done in order to better understand the mental health issues of this population. The objective was to follow up, get insight about the mental health symptoms of children scoring high on quantitative screening scales, and reassess children for clinical considerations and potential referrals to health care.

### Study tools in PROMISE SB

A structured questionnaire was administered to the parents during the PROMISE SB data collection. The questionnaire was developed in English, translated into the local language (Lumasaba), and back translated to English to ensure that the original meaning was retained. The word ‘parent’ was used to describe any caregiver or legal primary guardian, whether biological related or not.

### Selection of participants from PROMISE SB

The selection of children for the STC sub-study was done through screening tools in the PROMISE SB questionnaire, including the English (UK) parent version for children 4–17 years of the Strengths and Difficulties Questionnaire (SDQ) [29], and interviews with data collectors and study clinicians. The SDQ is a screening tool with 25 items related to five symptom scales—emotional, conduct, hyperactivity, peer, and prosocial. The target children were those with a total SDQ score of 14 or higher, thus including those with borderline scores (14–16) and those with abnormal scores (17–20) [29]. At the time of the data collection, we were not aware of any Ugandan-normed version of the SDQ with corresponding cut-off values, so our assumptions for borderline and abnormal scores were based on the original English (UK) version. However, a recent scoping review from Hoosen and colleagues points towards relatively frequent use of the SDQ in Africa, and they report on methodological challenges with corresponding implications for the interpretations of findings [30, 31]. We were aware of its usage in Uganda, and collegial feedback on its usefulness in that context suggested its content validity and its correlation with other mental health problems. The SDQ was thus chosen for the PROMISE SB, which had as its main outcome the detection of any ‘study-arm’ score difference [27, 30, 31]. In addition, the research personnel were interviewed regarding children whom they had identified as having social, emotional, and behavioural difficulties. The consultations with the personnel did not yield any additional cases to those found through screening with the SDQ.

The PROMISE SB aimed to see at least 70% of the 765 enrolled children participating in PROMISE EBF [28], in other words 535 children. We anticipated a psychiatric problem rate of approximately 20% [32] of the PROMISE-SB population, or around 110 cases. We also wanted to see cases with normal SDQ scores (< 14) for consistency and validity judgement. The cases scoring < 14 on the SDQ were consecutively recruited from PROMISE SB into the STC sub-study from June to December 2014. In total, the STC sub-study reassessed 120 children with high (≥ 14) SDQ scores and 29 children with low (< 14) SDQ scores (N = 149). The parents of the children were contacted to provide written informed consent before participation in the sub-study. Participants received a transport reimbursement and a meal for their participation.

### Diagnostic assessments

We used the Mini International Neuropsychiatric Interview for Children and Adolescents (MINI-KID) designed for children and adolescents aged 6–17 years to perform the mental health assessment of the selected children (N = 149). We used the full MINI-KID inventory and description, as described by Sheehan and colleagues in 2009 [33], to assess the presence of DSM-IV and ICD-10 psychiatric disorders in a comprehensive and concise way. The structured interview was held with the child and parent together. The MINI-KID interview has been shown to be useful in clinical and research settings [33], and it is regularly used clinically and in research in Uganda [34].

In this study, we report on the section on alcohol use in the MINI-KID. This section opens with three screening questions where a ‘yes’ on any of the following questions would open up for further questions: (1) whether the child ever had 3 or more drinks in a day; (2) whether the child ever had more than 3 drinks in 3 h; and (3) if this took place more than 3 times in the past year. The subsequent questions screened if alcohol was taken in a larger amount and probed for withdrawal symptoms, excessive drinking, unsuccessful efforts to cut down on drinking, time expenditure, additional problems in life due to drinking, hang-overs, reduced school performance, risk-taking, and legal problems. For positive responses on the alcohol section, comorbidities were assessed.

### Qualitative data description

The open-ended answers to the questionnaire were transcribed and sorted in a spreadsheet. The transcripts were coded on the following issues: (i) how the child got access to alcohol, particularly home brew versus commercially produced drinks; (ii) who provided the alcohol; (iii) the social circumstances; and (iv) the social implications.

### Quantitative data management and analysis

All completed questionnaires were entered into a database using Epidata (epidata.dk). The data were fitted with ranges and consistency checks. We report here the descriptive data on alcohol abuse and dependence and psychiatric comorbidity using the MINI-KID as described above.

### Ethics

Ethical approval was obtained from the Makerere University School of Medicine Research and Ethics Committee (SOM-REC ref. 2012-177) on 5 November 2012 and from the Uganda National Council for Science and Technology (Ref. SS 3123) on 22 April 2013. The sub-study extension was approved 11 July 2014. An additional written informed consent was obtained before the mental health interview commenced. A thumbprint was given in case of illiteracy, and the parents gave assent on behalf of their children. The psychiatric interviews were conducted by a psychiatrist specialising in child and adolescent psychiatry, and any critical finding from the interviews resulted in necessary counselling and referral.

## Results

Of the 765 mother-infant pairs recruited in Uganda into the PROMISE EBF trial [28], 530 (69.3%) parent–child pairs were included in the PROMISE SB study in Uganda for an interview on socio-demographic characteristics and for responding to the SDQ. Of the 530 parent–child pairs analysed in PROMISE SB [27], 124 children met the criterion for a mental health interview of an SDQ score ≥ 14, of whom 120 (96.0%) were participating in the STC sub-study. This was equivalent to 22.6% of the PROMISE SB cohort. We included 29/406 (7.1%) children with a total SDQ-score < 14. In total, we included 149 children, of whom 148 had complete data and were included in the analysis. Tables 1 and 2 show that the participants shared similar socio-demographic characteristics irrespective of being classified with high (≥ 14) or low (< 14) SDQ scores. There were relatively more boys in the group with a low SDQ score.

**Table 1:** Socio-demographic characteristics of STC sub-study participants by SDQ score, categorical variables

	SDQ ≥ 14 N = 119 N, %	SDQ < 14 N = 29 N, %
Sex of child		
Boy	63 (52.9)	20 (69.0)
Primary caregiver		
Mother	97 (81.5)	25 (86.2)
Father	4 (3.4)	1 (3.5)
Other	18 (15.1)	3 (10.3)
Caregiver marital status		
Caregiver married	89 (74.8)	18 (64.3)
Polygamous household	22 (18.5)	6 (20.7)
Literacy		
The father can read	93 (78.2)	19 (67.9)
The mother can read	77 (64.7)	17 (60.7)
Socio-economic status		
Socio-economic quintile		
Lowest	32 (26.9)	6 (20.7)
2^nd^	20 (16.8)	6 (20.7)
3^rd^	23 (19.3)	7 (24.1)
4^th^	19 (16.0)	3 (10.3)
Highest	25 (21.0)	7 (24.1)
Electricity	87 (73.1)	16 (55.2)
Fuel for cooking		
Wood	84 (70.6)	22 (76.9)
Coal	33 (27.7)	5 (17.2)
Drinking water		
Open source	46 (38.7)	11 (37.9)
Protected (tap, pump)	73 (61.3)	17 (58.6)
School and kindergarten attendance		
Never school or kindergarten	21 (17.6)	5 (17.2)
Either kindergarten or school	59 (49.6)	13 (44.8)
Prior kindergarten and current school	37 (31.1)	8 (27.6)
Allocated to the PROMISE EBF intervention	64 (53.8)	13 (44.8)

**Table 2:** Socio-demographic characteristics of STC sub-study participants by SDQ score, continuous variables

	SDQ ≥ 14 N = 119 Mean value (SD)	SDQ < 14 N = 29 Mean value (SD)
Child’s age, years	6.9 (0.6)	7.1 (0.7)
Mother’s age, years	33.4 (6.3)	33.4 (6.7)
Mother’s education, years	5.8 (3.0)	6.6 (3.2)
Father’s education, years	7.2 (3.5)	7.2 (3.1)
Number of people in household	8.1 (3.3)	6.9 (2.5)
Number of children of mother	5.1 (2.5)	5.6 (2.8)
Number of rooms in household	2.4 (1.3)	2.1 (1.0)
Months in kindergarten	16.3 (8.3)	17.9 (10.9)
Months in primary school	9.5 (6.1)	9.5 (6.1)
Height for age z-score	− 1.0 (1.1)	− 1.3 (0.9)

### Alcoholism in middle childhood

A total of 11 children were diagnosed with alcohol dependence (N = 6) and alcohol abuse (N = 5), of whom 10 had SDQ scores ≥ 14 (Table 3). The one child (1/29, 3.4%) with an SDQ score < 14 did not have any comorbidities. The 10 children with SDQ scores ≥ 14 had a variety of mental health comorbidities of which suicidality (3/10, 30.0%) and separation anxiety disorder (5/10, 50.0%) were the most common (Table 4). Their mean age at screening was 7.1 years (standard deviation 0.6 years).

**Table 3:** Alcohol dependence and abuse in the past 12 months according to DSM-IV criteria and combined by SDQ-group

Last 12 months	SDQ ≥ 14 N = 119 Mean value (SD)	SDQ < 14 N = 29 Mean value (SD)	Fisher exact test (1-sided)
Alcohol abuse	5 (4.2)	0	0.33
Alcohol dependence	5 (4.2)	1 (3.5)	0.67
Alcohol abuse and alcohol dependence combined	10 (8.4)	1 (3.45)	0.32

**Table 4:** Alcohol abuse and/or dependence and psychiatric co-morbidity

Alcohol abuse and/or dependence last 12 months	SDQ ≥ 14 N = 10*N, %
Major depressive episode	
Current	1 (10.0)
Past	1 (10.0)
Recurrent	1 (10.0)
Suicidality	3 (30%)
Panic disorder	1 (10.0)
Separation anxiety disorder	5 (50.0)
Specific phobia	1 (10.0)
Post-traumatic disorder	1 (10.0)
ADHD, inattentive	1 (10.0)
Conduct	1 (10.0)

*Out of 11 children abusing or being dependent on alcohol, 10 had an SDQ score ≥ 14. The one child with SDQ score < 14 did not have any co-morbidities

### The children’s comments

The 11 children shared stories of daily drinking and easy access through close family members, including through close relatives drinking themselves. While children primarily reported access through male relatives (fathers, grandfathers, or uncles), some also described access through female relatives, particularly if the women were involved in family brewing (mothers or grandmothers). Access to homemade brew was the most common source, as only one child reported that he was given purchased spirits. Thus, the families would have had at least some adults with knowledge of the underage drinking. A few of the children hid their drinking and got access through more distant relatives. Arriving to school late or leaving early from school in order to get access to alcoholic beverages was frequently reported. Many of the homes had stories of divorce, domestic violence, financial constraints, and violence. Some of the children complained of anxiety and trauma, such as witnessing violence and death, and some of the children used alcohol as ‘self-medication’ in their difficult circumstances, saying *“I feel good when I drink.”*

## Discussion

This study reports on the unexpected finding of alcohol abuse and dependence in middle childhood (5–8 years) in Mbale District, Uganda, among children living with their parents in their respective urban and rural communities. We describe the findings as ‘unexpected’ because we had discussed whether to even include the sections on drugs and alcohol in the MINI-KID assessment due to concerns around the relevance for the age group. We were surprised to find 11 out of 148 children with either alcohol dependence or abuse, and 10 of these children scored ≥ 14 on the SDQ. However, the sample size was far too small to perform statistical analysis on risk factors, population prevalence, or the magnitude of the problem.

We are worried that this might indicate the existence of a public health problem in Mbale District with underage drinking, not limited to the open use by street children, but also in the population of children living under family care. It is known that children experiment with alcohol worldwide. For example, in a prevalence study in Rwanda, the mean age of onset for substance use was 11.4 years [18], while a study among 8–10-year-old children in Argentina found that 58% had tasted alcohol, and approximately one third had tried alcohol several times [35]. The drinking in those studies occurred mostly under adult supervision, with care providers or other family members present [35]. Reviewing the literature on substance use in children below 10 years of age in low and lower middle-income countries (LLMIC), as defined by the World Bank [36], we only managed to identify three papers describing non-street-connected children, one from Kenya and two from Vietnam. These studies reported a prevalence of substance use of 1.3–13% [37–39]. A prevalence of 22.5–59.6% has been reported among street-connected children in LMIC [40, 41], including one study finding children initiating drinking as early as 5.5 years [42].

Our study did not investigate specific risk factors connected to alcohol abuse and dependence. As mentioned, before the study commenced we were unsure whether investigating substance use in this age group was even relevant. Although limited, our qualitative data do point to social vulnerability and poverty as driving factors for child alcoholism. This is in accordance with the finding that the prevalence of alcoholism is much higher among street-connected children, and not going to school was shown to be a significant risk factor among non-street-connected children [39].

All of the children in our sample had access to alcohol, but some of the children snuck away to drink and hid their drinking. Family history and peer influence have been found to be substantial contributors to the onset of alcohol use [20]. However, among the studies we identified investigating children under age 10 in LLMIC, the results were not as clear. A study from Kenya showed that living with a father, stepfather, or siblings increased lifetime drug use, but it found no significant correlation with the father’s substance abuse [38]. In two of the studies on children below age 10, no significant correlation was found with the father’s socio-economic status, household wealth, or family functioning [37, 38]. Peer use has been identified in one study in Vietnam as the strongest independent predictive factor for substance use in a sample of children. Having friends who drank was associated with a four-fold increase in risk for having tried substances [37]. Further investigations into the risk factors associated with childhood alcohol and substance use are warranted.

Although our study did not investigate specific risks or associated factors such as socio-economic, parenting or peer-factors, our study seems to indicate a relationship between mental health challenges and alcohol use among the very young. This is well established in older age groups [22]. Our findings may suggest a vicious cycle where alcohol drinking leads to less acceptable behaviour, more social stigmatisation, and more emotional stress. However, we also saw that alcohol was used as a strategy to relieve stress expressed as “I feel good when I drink.”

Our study has clear limitations. First, the study was not designed to estimate prevalence or risk factors or to validate the findings of the study. It is therefore important to investigate childhood drinking in a more systematic manner in the region. Secondly, we focused the reassessment on those scoring high on the SDQ, resulting in a biased sample. The group of children with lower scores on SDQ was limited to only 29/406, so we were not able to make any conclusions about that group. Also, the chosen cut-offs were based on UK thresholds, and those have not been normed for Uganda. Further, the available MINI-KID instrument at the time of the study was based on the DSM-IV criteria for alcohol abuse and dependence, but in the updated DSM-5 these are now classified together as alcohol use disorder and are categorised into mild, moderate, and severe. Thus, we are not able to suggest how our findings might correspond to the new diagnostic classification. Further, the MINI-KID screening questions as given in the method section might not be appropriate for very young children. For example, the thresholds of three drinks in a day or three drinks in three hours are high and might suffer from subjective interpretation. First, fewer than three drinks did not signal a positive answer on the screening, and this might also represent a problem. Children might also have problems defining the drinks, both in quantity and frequency. This is particularly true if they are drinking from drinking pots, using small sachets, or taking sips from leftover drinks. Thus, we are worried that our findings most likely highly underestimate the problem. Despite these limitations, we believe it is justified to share these findings because they describe a possible severe public health issue in need of further investigation.

## Conclusions

Our findings show that while the magnitude is not yet established, alcohol use is occurring in children aged 5–8 years in Mbale District, Uganda. This finding should have implications for future studies that investigate the prevalence of alcohol use disorders and related risk factors and outcomes in similar settings. Meanwhile, health workers and educators working with this age group should not assume the non-existence of these conditions.

## Data Availability

The PROMISE SB consortium host institution Makere University, Uganda is legally bound to make the data available through the Saving Brains Project implementation committee. To request data access, please email savingbrainsimplementationcom@chs.mak.ac.ug.

## Abbreviations

DSM: Diagnostic and Statistical Manual of Mental Disorders
ICD-10: International Classifications of Diseases
LLMIC: Low and Lower Middle-Income Countries
LMIC: Lower Middle-Income Countries
MINI-KID: Mini International Neuropsychiatric Interview for Children and Adolescents
PROMISE EBF: Promoting exclusive breastfeeding for six months by peer counsellors in Burkina Faso, Uganda, and South Africa
PROMISE SB: Saving Brains in Uganda and Burkina Faso
SDQ: Strengths and Difficulties Questionnaire
STC: SeeTheChild –Mental Child Health in Uganda
YLD: Years Lived with Disability
WHO: World Health Organisation
